# X-Ray of One-Sided “White Lung” after Central Venous Catheterization

**DOI:** 10.1155/2014/320264

**Published:** 2014-01-19

**Authors:** Michel Casanova, Wolfgang Ummenhofer

**Affiliations:** Department for Anesthesia, Surgical Intensive Care, Prehospital Emergency Medicine and Pain Therapy, University Hospital of Basel, Spitalstrasse 21, 4031 Basel, Switzerland

## Abstract

Complications during insertion of a subclavian central venous line are rare but potentially serious. This case report describes the radiological abnormality of a one-sided pleural effusion during a routine control directly after a difficult central venous catheterization. We illustrate the findings, the initial emergency management, and our procedure to rule out an iatrogenic hemothorax. Possible differential diagnoses and strategies for management of a suspected complication are discussed.

## 1. Introduction

For patients in the United States, as many as five million central venous lines are placed annually.

Perforation of the subclavian artery occurs in about 0.1–1% of cases, leading to hemothorax (1%) and rarely quadriplegia. Perforation of the aorta and cardiac tamponade can occur if the cannula-site perforation is within the pericardial reflection. This complication is associated with a death rate of 90% [1, 2]. Compulsory radiological control of central venous access is debated but is still routinely performed in our institution.

## 2. Case Description

An 88-year-old woman was initially admitted for correction of poorly controlled diabetes mellitus. During her hospitalization, she developed peripheral catheter-associated *Staphylococcus aureus* thrombophlebitis in her left cubital region. The patient was on IV antibiotics for four days. Initially, trimethoprim/sulfamethoxazole had been used. After 2 days, this was followed by ceftriaxone due to antibiotic resistance. After deciding on long-term antibiotic treatment with IV cefazolin and due to bad peripheral venous conditions, the patient was scheduled for central venous catheterization.

Relevant diagnoses of the patient were (i) known hypertensive cardiac disease (transthoracic echocardiography 6 months before had shown a LV-EF of 54% and a light mitral and tricuspid insufficiency) and (ii) liver cirrhosis with portal hypertension.

A recent CT scan showed signs of liver cirrhosis with ascites and splenomegaly, and abdominal sonography 2 months ago supposed the findings to be due to a steatohepatitis. Liver serologies were negative for a lack or deficiency of alpha-1 antitrypsin. Ceruloplasmin and alpha-fetoprotein were normal. No signs of active viral hepatitis were found, and the patient's carcinoembryonic antigen value was within the normal range. Finally, cytological screens of the ascites showed no signs of malignancy.

Besides the antibiotics, medical therapy at the time of consultation included torsemide, spironolactone, and sitagliptin.

Laboratory examination revealed thrombocytopenia (78,000/*μ*L) and a spontaneous international normalized ratio INR of 1.2. The thrombocytopenia was assumed to be due to splenomegaly (Table 1).

Clinically, the patient breathed normally and had a normal heart rate and blood pressure when arriving for the intervention. For comfort reasons of the patient, we decided to perform cannulation of the right subclavian vein. Cannulation of the vein was successful upon the first attempt, but we were twice unable to advance the guide wire. Consequently, we changed to an ultrasound-controlled puncture of the right internal jugular vein, which worked well on the first attempt. The catheter was placed without problem. According to our internal guidelines, a chest X-ray was taken on the patient's return to the ward.

**Figure 1:**
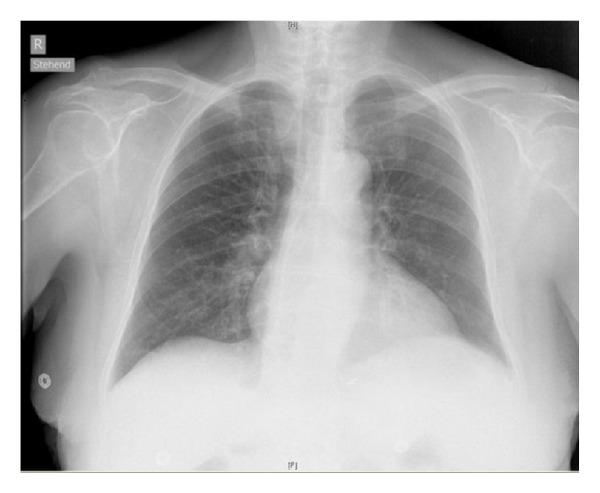
Chest X-ray anteroposterior at admission (sitting).

**Figure 2:**
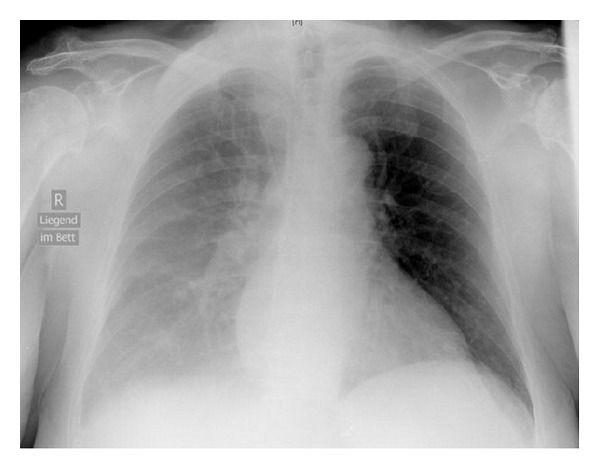
Chest X-ray anteroposterior after puncture (lying).

Surprisingly, when controlling the radiographic finding one hour after the procedure, the X-ray showed a one-sided “white lung.” Comparison of the chest X-ray from the day of admission showed no effusion or cardiac decompensation (Figure 1), whereas the current image clearly depicted what we believed to be a pleural effusion on the right side, which was the puncture site for the subclavian as well as the internal jugular vein (Figure 2).

We were suspicious of an iatrogenic lesion of the right subclavian artery or vein with consecutive hematothorax and immediately saw the patient at the ward.

Contrary to our apprehension, we found the patient to be hemodynamically stable and without dyspnea. Auscultation of the right chest revealed breath sounds but percussion of the right hemithorax an obvious dullness. In addition, a bedside HemoCue measurement showed a lowered hemoglobin value of 96 g/L compared with 130 g/L the day before.

We took specimens for type and screening, blood cell count, and emergency chemistry and reflected on reasonable next steps and possible options. Of course, a chest drain would have relieved possible hemothorax, but the lack of hemodynamic or respiratory compromise did not require immediate action. With strong suspicion of arterial or venous perforation, we felt that a CT scan could be helpful and that additional perfusion CT could be an important asset for localization of the potential bleeding site. Internal stenting and/or surgical repair would probably benefit from radiological identification of the possible iatrogenic injury.

**Figure 3:**
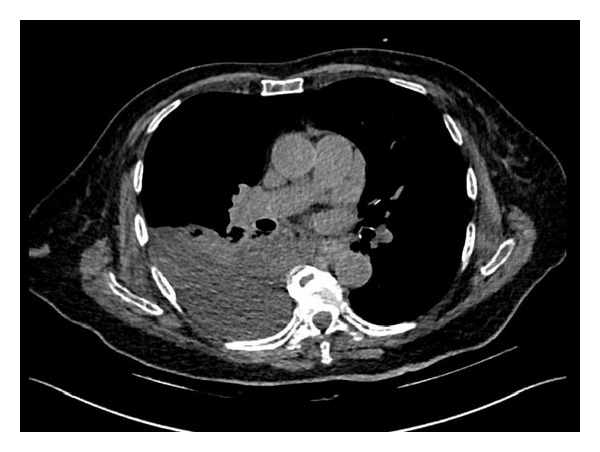
CT scan (MDCT, arterial with 40 mL Ultravist 370 and 5 min p.i. DLP 335 mGycm) of the thorax.

We involved the CT consultant on call, who confirmed the plausibility of the indication and initiated a plain and subsequent contrast medium CT scan (MDCT, arterial with 40 mL Ultravist 370 and 5 min pi; DLP 335 mGycm). Fortunately, no arterial or venous leakage with active bleeding could be found. In addition and even more surprisingly, calculation of density revealed that a hemothorax was unlikely to be the source of the white chest X-ray. The Hounsfield scale value of 0 HU native of the liquid was consistent for an effusion (Figure 3), and the preliminary CT diagnosis was a right pleural effusion 6 cm long.

**Figure 4:**
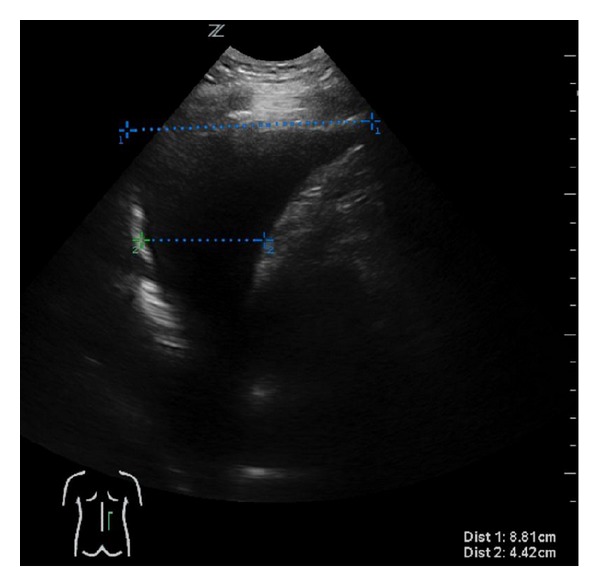
Ultrasound of lower thorax.

A pleural sonograph (Figure 4) showed an estimated 900 mL of homogenic anechogenic pleural effusion. The subsequent ultrasound-guided puncture extracted clear liquid, and the hematothorax could be definitively ruled out.

The evaluation of the laboratory values of the puncture liquid showed a transudate according to Light's criteria (Table 2) [3]. No signs of mycobacteria could be found.

We received the above-mentioned emergency laboratory (Table 1), which showed a hemoglobin value of 120 g/L in comparison to 130 g/L the day before. Retrospectively, the HemoCue bedside value seemed to be an inaccurately low determination.

The conclusive diagnosis for our patient was a right-sided pleural effusion due to a nonalcoholic steatohepatitis (NASH)-mediated liver cirrhosis. The patient had already suffered a right-sided pleural effusion five months earlier, which was unknown to both us and the treating physician at the ward at the time our intervention. Treatment for the ascites was adjusted. Spironolactone and torsemide were augmented, and lactulose was added.

## 3. Discussion

Routine chest X-ray following central venous catheterization revealed a white chest on the puncture side. The position of the catheter tip was radiologically correct. However, the puncture process with unsuccessful attempt for subclavian vein access (impossibility to insert the guide wire and a second puncture site at the internal jugular vein of the same side) aroused suspicion for a perforation complication and a subsequent hemothorax. Since a chest X-ray few days before our intervention had been normal, we were even more concerned.

However, immediate clinical examination could exclude acute hemodynamic and respiratory compromise. Interdisciplinary discussion of options with radiology and pneumology consultants offered a reasonable strategy for an eventually unexpected explanation.

Besides a hemothorax, there are more than 50 recognized causes of one-sided pleural liquid accumulation. Pleural effusions are a common medical problem resulting from diseases local to the pleura or underlying lung, systemic conditions, organ dysfunction, and drugs [4]. In addition, different etiologies can produce one-sided effusions more frequently than others.

In cardiac insufficiency, bilateral effusions are present in 81%, right-sided in 12%, and left-sided in 7% of all cases [5]. Hepatic hydrothorax is a rare but important cause of—usually unilateral—pleural effusion. About 85–90% of the cases are isolated right-sided effusions [6]. Hepatic hydrothorax can be observed as a complication of portal hypertension in <10% of patients with ascites secondary to advanced liver cirrhosis. The underlying pathophysiological mechanism seems to be a direct movement of fluid from the peritoneal cavity into the pleural space through diaphragmatic defects [7]. In rare cases, complete translocation of fluid into the pleural space without ascites can be observed [8, 9].

## Figures and Tables

**Table 1:** Summary of important laboratory values.

	Measured	Normal value
Hematology		
Hemoglobin (g/L)	120	140–180
Hematocrit (%)	34	40–52
Thrombocytes (×10^9^/L)	78	136–380
Chemistry		
INR	1.2	0.8–1.2
Creatinine (Nmol/L)	164	81–133
Albumin (g/L)	23	35–53
Bilirubin total (Nmol/L)	11	5.0–18.0
CRP (mg/L)	27.5	<5.0
ASAT (U/L)	40	11.0–34.0
ALAT (U/L) 45 8–63	24	8.0–41.0
Alkaline phosphatase (U/L)	168	<104
GGT (U/L)	278	6.0–40.0
Total protein (g/L)	62	64–83
LDH (U/L)	209	135–214
Pleural punctate		
Total protein (g/L)	16	
LDH (U/L)	50	
Bacterial growth	No aerobic- or anaerobic bacterial growth	
Gram staining	No bacteria	
Mycobacterium-tuberculosis complex PCR	Negative	

ALAT: alanine transaminase; ASAT: aspartate transaminase; CRP: C-reactive protein; GGT: gamma-glutamyltransferase; INR: international normalized ratio; LDH: lactate dehydrogenase.

**Table 2:** Light's criteria [3].

Pleural fluid is an exudate if one or more of the following criteria
are met
(i) Pleural fluid protein divided by serum protein is > 0.5
(ii) Pleural fluid lactate dehydrogenase (LDH) divided by serum
LDH is > 0.6
(iii) Pleural fluid LDH > 0.6 or is 2/3 times the normal upper limit
for serum
